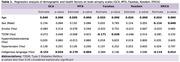# Association between brain structure imaging and health factors in a low education cohort

**DOI:** 10.1002/alz70860_107741

**Published:** 2025-12-23

**Authors:** Gregory Brown, Diego Bustamante‐Paytan, José Carlos Huilca, Maria Fe Albujar‐Pereira, Katherine Aguero, Graciet Verastegui, Zadith Yauri, Pamela Bartolo, Daniela Bendezu, Karol Melissa Lipa‐Pari, Rosa Montesinos, Nilton Custodio

**Affiliations:** ^1^ Instituto Peruano de Neurociencias, Lima, Lima, Peru; ^2^ University of California, San Francisco, San Francisco, CA, USA; ^3^ Unidad de Investigación de Deterioro Cognitivo y Prevención de Demencia, Instituto Peruano de Neurociencias, Lima, Lima, Peru; ^4^ Unidad de Investigación de Deterioro Cognitivo y Prevención de Demencia, Instituto Peruano de Neurociencias, Lima, Peru, Lima, Lima, Peru; ^5^ Unidad de Investigación de Deterioro Cognitivo y Prevención de Demencia, Instituto Peruano de Neurociencias, Lima, Peru; ^6^ Hospital Nacional Cayetano Heredia, Lima, Lima, Peru; ^7^ Universidad de San Martín de Porres, Facultad de Medicina, Centro de Investigación del Envejecimiento, Lima, Lima, Peru; ^8^ Unidad de Investigación y Docencia, Equilibria, Lima, Peru., Lima, Lima, Peru; ^9^ Equilibria, Lima, Lima, Peru

## Abstract

**Background:**

Dementia is rising globally, with a significant impact in Latin America. Low education has been associated with reduced cognitive reserve and structural brain changes, increasing vulnerability to neurodegeneration. However, the factors influencing these brain alterations remain an emerging area of research. This study aims to evaluate the association between brain atrophy scales and health factors in a low‐education Peruvian cohort to better understand neuroprotection and vulnerability in this population.

**Method:**

This study was conducted on cognitively healthy individuals with low education (<7 years) at the Instituto Peruano de Neurociencias, Lima, Peru. Non‐contrast brain MRIs were performed to assess global cortical atrophy (GCA), medial temporal lobe atrophy (MTA), Koedam score, ERICA score, and Fazekas scale. Scores were averaged between an expert dementia neurologist and a neuroradiologist. Both evaluations and final scores were conducted independently and blinded. Linear regression was used to assess the association between health factors and MRI metrics. IRB approval was obtained for the study.

**Result:**

A total of 205 participants were included in the study. Knowledge of an Indigenous language (Quechua or Aymara) was identified as a neuroprotective factor for GCA, MTA, and ERICA (*p*‐values: 0.024, 0.005, and 0.014, respectively). Patients with diabetes mellitus type 2 scored lower on the Fazekas scale, indicating fewer white matter hyperintensities (*p*‐value: 0.039). No significant effects were observed for smoking, hypercholesterolemia, or hypertension.

**Conclusion:**

These findings suggest a potential neuroprotective role of Indigenous language knowledge on cortical and medial temporal atrophy. The unexpected association between diabetes and lower WMH requires further investigation. The absence of significant effects from vascular risk factors highlights the need for further studies to explore determinants of brain structure in low‐education populations.